# In-Depth Investigation of the Safety of Wooden Shelves Used for Traditional Cheese Ripening

**DOI:** 10.1128/AEM.01524-21

**Published:** 2021-11-10

**Authors:** Luca Settanni, Gabriele Busetta, Valeria Puccio, Giuseppe Licitra, Elena Franciosi, Luigi Botta, Rosalia Di Gerlando, Massimo Todaro, Raimondo Gaglio

**Affiliations:** a Dipartimento Scienze Agrarie, Alimentari e Forestali, Università di Palermo, Palermo, Italy; b Dipartimento di Agricoltura, Alimentazione e Ambiente (Di3A), Università degli Studi di Catania, Catania, Italy; c Research and Innovation Centre, Fondazione Edmund Mach (FEM), San Michele all’Adige, Italy; d Dipartimento di Ingegneria, UdR INSTM di Palermo, Università degli Studi di Palermo, Palermo, Italy; University of Helsinki

**Keywords:** cheese ripening, lactic acid bacteria, MiSeq Illumina, scanning electron microscopy, traditional cheeses, wooden shelves

## Abstract

The main goal of this research was to characterize the bacterial diversity of the wooden boards used for aging traditional Sicilian cheeses and to evaluate whether pathogenic bacteria are associated with these surfaces. Eighteen cheese dairy factories producing three traditional cheese typologies (PDO Pecorino Siciliano, PDO Piacentinu Ennese, and Caciocavallo Palermitano) were selected within the region of Sicily. The wooden shelf surfaces were sampled by a destructive method to detach wood splinters as well as by a nondestructive brushing to collect microbial cells. Scanning electron microscopy showed the presence of almost continuous bacterial formations on the majority of the shelves analyzed. Yeasts and fungal hyphae were also visualized, indicating the complexity of the plank communities. The amplicon library of the 16S rRNA gene V3-V4 region was paired-end sequenced using the Illumina MiSeq system, allowing the identification of 14 phyla, 32 classes, 52 orders, 93 families, and 137 genera. Staphylococcus equorum was identified from all wooden surfaces, with a maximum abundance of 64.75%. Among cheese-surface-ripening bacteria, *Brevibacterium* and *Corynebacterium* were detected in almost all samples. Several halophilic (*Halomonas*, Tetragenococcus halophilus, *Chromohalobacter, Salimicrobium*, *Marinococcus*, *Salegentibacter*, *Haererehalobacter*, *Marinobacter*, and *Idiomarinaceae*) and moderately halophilic (*Salinicoccus*, *Psychrobacter*, and *Salinisphaera*) bacteria were frequently identified. Lactic acid bacteria (LAB) were present at low percentages in the genera *Leuconostoc*, *Lactococcus*, *Lactobacillus*, *Pediococcus*, and Streptococcus. The levels of viable microorganisms on the wooden shelves ranged between 2.4 and 7.8 log CFU/cm^2^. In some cases, LAB were counted at very high levels (8.2 log CFU/cm^2^). Members of the *Enterobacteriaceae* family were detected in a viable state for only six samples. Coagulase-positive staphylococci, Salmonella spp., and Listeria monocytogenes were not detected. Seventy-five strains belonged to the genera *Leuconostoc*, *Lactococcus*, *Pediococcus*, *Enterococcus*, *Lactobacillus*, and *Weissella*.

**IMPORTANCE** This study provides evidence for the lack of pathogenic bacteria on the wooden shelves used to ripen internal bacterially ripened semihard and hard cheeses produced in Sicily. These three cheeses are not inoculated on their surfaces, and surface ripening is not considered to occur or, at least, does not occur at the same extent as surface-inoculated smear cheeses. Several bacterial groups identified from the wooden shelves are typically associated with smear cheeses, strongly suggesting that PDO Pecorino Siciliano, PDO Piacentinu Ennese, and Caciocavallo Palermitano cheese rind contributes to their final organoleptic profiles.

## INTRODUCTION

Several traditional Italian cheeses are ripened on wooden shelves. In Sicily, the entire cheese production process is performed with wooden equipment for all typical products (1–3), and the contact with wood begins when milk is collected in the wooden vats used for curdling (4–6). Up to date, there has been no specific contraindication to the use of wood as a food contact material, because European Regulation (EC) no. 1935/2004 regarding the materials in contact with foods does not refer to wood (7). To this purpose, member countries legislate at different levels (8), and the basic principle is that any material used for preparation, packaging, and wrapping of foods must not transfer its constituents to foods (9). For this reason, dairy production in Italy can be carried out with wooden equipment under EC no. 2074/2005, which allows derogation from EC no. 852/2004 for foods with traditional characteristics (9).

In order to demonstrate the suitability of wood for dairy purposes, different French and Italian research groups focused on the microbial characterization of wooden vats, showing how lactic acid bacteria (LAB), responsible for curd acidification (starter LAB) and cheese ripening (non-starter LAB), form stable biofilms (4, 10–13). The same authors also evaluated the presence of Escherichia coli and coagulase-positive staphylococci as process hygiene criteria and Salmonella spp. and Listeria monocytogenes as food safety criteria in light of the Commission Regulation (EC) no. 2073/2005 (14), finding that while coliforms were counted at very low densities, the other pathogenic bacteria were never detected. The absence of the undesired bacteria in the wooden vats was better investigated by Cruciata et al. (15), who contaminated raw milk with the four main dairy pathogens and, after cheese making, showed that the neoformed LAB biofilms on the wood surfaces prevented their attachment to the vats. The research on the safety aspects of the wooden shelves is more limited; to our knowledge, only Mariani et al. (16) have performed microbial characterization of wooden shelves used for the ripening of French Reblochon de Savoie smear cheeses. By a culture-dependent approach, the authors found that micrococci and corynebacteria, yeasts, and molds constituted the dominant microflora on the shelves, while leuconostocs, lactobacilli, enterococci, staphylococci, and pseudomonads were found at low levels. However, smear cheeses are generally inoculated by commercially available surface starters (17), and this might explain the dominance of micrococci and corynebacteria and yeasts found on the shelves by Mariani et al. (16). The same research group evaluated also the inhibition of L. monocytogenes growth by the wooden shelf biofilms, indicating a certain potential of wood for cheese bioprotection against food pathogens (18).

In the last 15 years, the application of culture-independent techniques to study the microbial communities of complex matrices, including cheeses, increased consistently (19, 20). Several reasons explain the success of culture-independent approaches. First, culture-based techniques unavoidably underestimate the microbial diversity of a given matrix since the subdominant species present can be outcompeted in culture by numerically abundant species. Sometimes, culture-dependent techniques can even fail to detect some dominant microbial groups (20, 21). Second, the modern high-throughput sequencing (HTS) of DNA of all microbial nucleic acids (shotgun sequencing) present in a given cheeses provides a complete overview of the microbial community (22). Regarding wooden shelves used in cheese production, Guzzon et al. (23) applied 454 pyrosequencing to study the biofilms hosted onto the planks for smear Fontina cheese ripening, finding a cause-effect relationship between the dominant *Actinobacteria* and the red-brown pigmentation defect.

Based on the above considerations, the present work was carried out to characterize the wooden shelves used for the ripening of three typical and traditional cheese typologies (PDO Pecorino Siciliano, PDO Piacentinu Ennese, and Caciocavallo Palermitano cheeses) produced in Sicily by a combined culture-dependent and HTS approach. The microbial biofilms were microscopically investigated by scanning electron microscopy (SEM) before being analyzed by MiSeq Illumina technology and subjected to plate counts. The dominant bacteria were phenotypically and genetically grouped and identified.

## RESULTS

### Scanning electron microscopy of wooden shelf biofilms.

**FIG 1:**
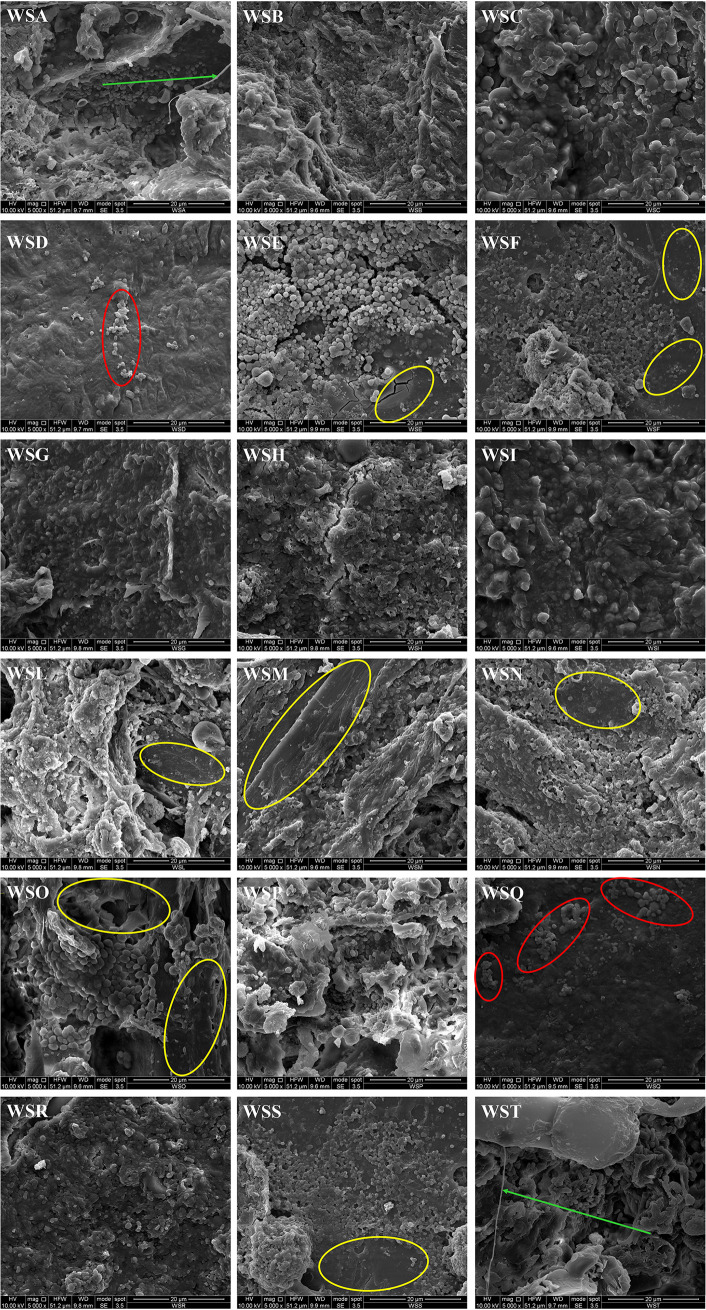
Scanning electron microscopy observations of wooden splinters collected from wooden shelves used to ripen PDO Pecorino Siciliano, PDO Piacentinu Ennese, and Caciocavallo Palermitano cheeses in 18 dairy facilities. Abbreviations: WSA to -T, wooden shelves from factories A to T, respectively. Red ovals highlight limited bacterial aggregations, yellow ovals highlight the woody roughness of the board surfaces, and green arrows indicate fungal hyphae.

The photographs taken during SEM analysis of wood splinters collected from the wooden shelves used to ripen PDO Pecorino Siciliano, PDO Piacentinu Ennese, and Caciocavallo Palermitano cheeses in 18 dairy facilities are reported in Fig. 1. Although the majority of shelves were characterized by continuous microbial attachments, some samples such as those from WSD (i.e., wooden shelf from factory D) and WSQ showed only a few cells. Also, when the biofilms covered almost the entire surface of the wooden shelves, some woody roughness is clearly visible as per the WSE, WSF, WSL, WSM, WSN, WSO, and WSS samples. However, unlike the wooden vats used for milk curdling, the wooden shelves used for cheese ripening were not generally characterized by the extracellular polysaccharide structures typical of bacterial biofilms. SEM inspection was useful to recognize cocci and rods and to predict their proportions. In particular, the majority of biofilms were dominated by coccus-shaped populations, but the shelves WSG, WSH, WSN, and WSR showed a consistent presence of short rod bacteria. Yeast cells were frequently detected, especially for the shelves WSC, WSI, and WSO and a few fungal hyphae were also found (samples from WSA and WST), confirming the complexity of the microbial community of the shelves used to ripen traditional Sicilian cheeses.

### Taxonomic distribution of wooden shelf bacteria.

**FIG 2:**
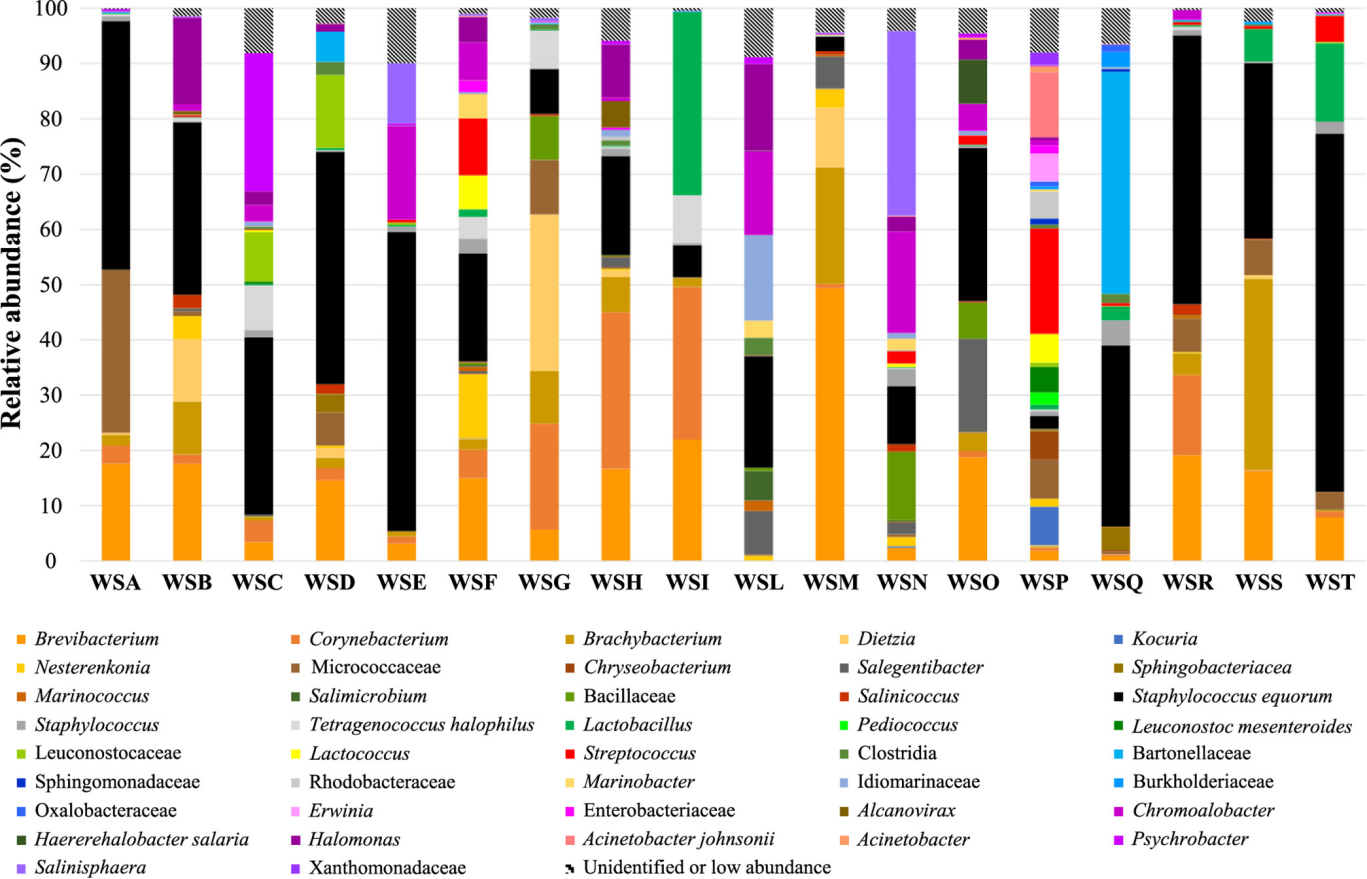
Relative abundances (%) of bacteria identified by MiSeq Illumina from the biofilms on the wooden shelves used to ripen PDO Pecorino Siciliano, PDO Piacentinu Ennese, and Caciocavallo Palermitano cheeses in 18 dairy facilities. The amplification occurred on a 464-nucleotide sequence of the V3-V4 region of the 16S rRNA gene. Only taxa occurring at >0.1% abundance in at least one sample were included. Abbreviations: WSA to -T, wooden shelves from factories A to T, respectively.

The extracted DNA was always successfully amplified in the bacterial V3-V4 16S rRNA gene region. A total of 954,257 paired-end sequences had been obtained. The taxonomy classification allowed to identify 14 phyla, 32 classes, 52 orders, 93 families, and 137 genera. Figure 2 shows the relative abundance (%) of the operational taxonomy units (OTUs) identified in the biofilms collected from the wooden shelves sampled. The OTUs with an individual relative abundance below 0.1% were not considered since this is the threshold indicated for abundant communities (24).

All wooden shelves hosted Staphylococcus equorum in abundances that ranged from 2.28% of the OTUs of the sample from WSP to 64.75% of those of WST. Among other staphylococci, not detected in WSM, their abundance did not exceed 4.54% (in WSQ), and the abundance was below 1% in the majority of shelves. *Micrococcaceae* were not consistently present in most of the shelves, but detected at very high levels in WSA. Within this family, *Kocuria* was detected in two samples, from WSN and WSP, at 0.29 and 6.95%, respectively. Other bacterial groups associated with surface ripening, such as *Brevibacterium* and *Corynebacterium*, were identified in almost all wooden shelves, except WSL (negative for both genera) and WSN (negative for *Corynebacterium*). Their incidence among the wooden shelf biofilms was quite variable: basically below 10% for both in most of the samples, but *Brevibacterium* OTUs accounted for almost 50% in WSM biofilm. Except for three shelves, *Brachybacterium* was detected in all other samples at very high percentages in WSB, WSG, WSM, and WSS biofilms. A similar trend was observed for *Bacillaceae*.

Among halophilic or halotolerant species, *Halomonas* was mainly found on the shelves WSB, WSH and WSL, Tetragenococcus halophilus on WSC, WSG, and WSI, and *Chromohalobacter* on WSE, WSF, WSL, and WSN. The halophilic groups *Salimicrobium*, *Marinococcus*, *Salegentibacter*, *Haererehalobacter*, *Marinobacter*, and *Idiomarinaceae* and the moderately halophilic *Salinicoccus*, *Psychrobacter*, and *Salinisphaera* were found in a few shelves, and their abundance was particularly variable.

Besides the halophilic tetragenococci, the LAB community of the wooden shelves was represented by the *Leuconostoceae* family, with Leuconostoc mesenteroides the only species identified, as well as *Lactococcus*, *Lactobacillus*, *Pediococcus*, and Streptococcus. *Lactobacillus* was basically present at very low levels, even though in samples WSI and WST, the *Lactobacillus* OTUs represented 33.12 and 14.18%, respectively. Pediococci and leuconostocs were detected at low percentages in a few samples, only the wooden shelf WSP showed their presence at 2.20 and 5.46%. Similar findings were observed for lactococci and streptococci, generally identified in a few samples at low concentrations, but the shelves WSF, WSP, and WST showed their abundance between almost 5 and 20%.

The other bacterial groups showed an overall low relative abundance, but in one of very few samples, their presence was found at particularly high percentages, like *Bartonellaceae* reaching 40.13% in WSQ, *Dietzia* with 28.36% in WSG, and *Nesterenkonia* with 11.60% in WSF. Members of the *Enterobacteriaceae* family were detected at very low abundances in only four wooden shelves. Acinetobacter johnsonii was found only in two samples, but in one case (WSP), its abundance was 11.79%. Other acinetobacters were detected in WSO and WSP biofilms at 0.36 and 1.09%, respectively. WSP biofilm also hosted 5.13% *Chryseobacterium*, which was also found at very low levels in the WSQ sample. The last two shelves included *Sphingomonadaceae* among their communities. *Sphingobacteriaceae* were found not only in WSP and WSQ biofilms, but also on the surface of WSD, WSH, and WSR shelves at low percentages.

Clostridia were found in several biofilms, but their percentages were quite low: the highest level (3.19%) was displayed by the WSL sample. A low incidence of *Rhodobacteriaceae*, *Burkholderiaceae*, *Oxalobacteriaceae*, *Xanthomonadaceae*, and *Erwinia* was found in a few samples. *Alcanivorax* was detected at consistent levels (4.81%) only in WSH biofilm and was also found in SWB at very low levels (0.73%).

### Levels of viable microorganisms.

The results of the plate counts of the main food microbial groups are reported in Table 1. According to Tukey’s test, statistically significant differences (*P* < 0.0001) were found for the levels of all microbial groups that were the objects of investigation. Total mesophilic microorganisms (TMM) ranged between 2.4 and 7.8 log CFU/cm^2^, indicating a consistent numerical difference among the 18 shelves analyzed. The lowest loads were enumerated for WSF, used for 13 years, while the youngest shelf (WSO), barely used for 1 year, displayed TMM levels of 6.1 log CFU/cm^2^. The cell densities were highly variable on the different wood types. On average, TMM were 5.0, 5.1, and 5.8 log CFU/cm^2^ on the shelves used for PDO Piacentinu Ennese, Caciocavallo Palermitano, and PDO Pecorino Siciliano cheeses, respectively.

**TABLE 1:** Microbial loads^*a*^ of wooden shelf biofilms

Sample^*a*^	Bacterial count (log CFU/cm^2^) of^*b*^:											
	TMM	*Enterobacteriaceae*	Total coliforms	E. coli	Pseudomonads	Enterococci	Rod LAB		Coccus LAB		Yeasts	Molds
							MRS at 30°C	MRS at 44°C	M17 at 30°C	M17 at 44°C		
WSA	6.1 ± 0.2 BCD	2.1 ± 0.2 C	0 D	<1 D	3.8 ± 0.3 BC	2.2 ± 0.2 DEF	4.1 ± 0.2 DEF	0 H	6.2 ± 0.2 BC	1.5 ± 0.1 F	3.7 ± 0.2 GH	<1 G
WSB	5.8 ± 0.3 CDE	0 E	0 D	<1 D	5.7 ± 0.4 A	2.2 ± 0.1 DEF	4.8 ± 0.4 BCD	2.3 ± 0.1 E	6.0 ± 0.5 BCD	1.6 ± 0.2 F	2.7 ± 0.2 IJ	1.8 ± 0.1 F
WSC	6.0 ± 0.2 BCD	4.9 ± 0.3 A	4.3 ± 0.3 A	4.7 ± 0.3 A	5.8 ± 0.3 A	2.6 ± 0.3 CDE	5.3 ± 0.5 B	3.2 ± 0.2 CD	5.6 ± 0.4 BCD	3.0 ± 0.2 D	3.4 ± 0.4 HI	2.0 ± 0.3 EF
WSD	5.0 ± 0.2 EF	0 E	0 D	<1 D	<1 E	2.0 ± 0.2 EFG	3.0 ± 0.2 GH	1.1 ± 0.1 G	5.0 ± 0.4 D	1.8 ± 0.3 F	2.1 ± 0.2 JK	1.8 ± 0.2 F
WSE	6.3 ± 0.2 BC	2.0 ± 0.2 C	0 D	<1 D	3.3 ± 0.2 C	1.1 ± 0.1 I	3.9 ± 0.3 EF	1.7 ± 0.2 F	6.5 ± 0.5 B	6.0 ± 0.4 A	6.3 ± 0.2 AB	2.1 ± 0.2 EF
WSF	2.4 ± 0.1 H	0 E	0 D	<1 D	<1 E	1.9 ± 0.2 EFGH	1.3 ± 0.1 J	0 H	1.6 ± 0.2 F	1.3 ± 0.2 F	1.8 ± 0.2 K	2.6 ± 0.3 E
WSG	5.5 ± 0.3 CDE	0 E	0 D	<1 D	<1 E	1.5 ± 0.2 FGHI	4.3 ± 0.2 CDE	0 H	5.2 ± 0.3 CD	1.8 ± 0.2 F	4.8 ± 0.3 DEF	4.9 ± 0.4 AB
WSH	7.8 ± 0.4 A	3.6 ± 0.2 B	3.3 ± 0.2 B	3.5 ± 0.2 B	5.2 ± 0.3 A	4.1 ± 0.4 A	8.2 ± 0.3 A	4.2 ± 0.2 A	8.1 ± 0.3 A	4.4 ± 0.3 B	6.4 ± 0.2 AB	4.8 ± 0.3 AB
WSI	6.8 ± 0.2 B	0 E	0 D	<1 D	4.3 ± 0.2 B	1.3 ± 0.1 GHI	5.3 ± 0.3 B	0 H	5.9 ± 0.3 BCD	0 G	6.8 ± 0.1 A	5.4 ± 0.4 A
WSL	5.3 ± 0.4 DE	1.3 ± 0.1 D	1.2 ± 0.1 C	<1 D	4.0 ± 0.2 B	3.5 ± 0.3 AB	3.4 ± 0.2 FG	3.4 ± 0.3 CD	6.6 ± 0.4 B	4.0 ± 0.2 BC	5.2 ± 0.3 CDE	<1 G
WSM	5.4 ± 0.3 DE	0 E	0 D	<1 D	4.3 ± 0.3 B	1.6 ± 0.1 FGHI	1.9 ± 0.1 IJ	1.5 ± 0.2 FG	5.8 ± 0.3 BCD	1.8 ± 0.1 F	5.4 ± 0.3 CD	3.4 ± 0.3 D
WSN	4.2 ± 0.3 FG	0 E	0 D	<1 D	3.3 ± 0.2 C	3.5 ± 0.4 AB	3.5 ± 0.3 EFG	3.5 ± 0.3 BC	3.8 ± 0.4 E	3.4 ± 0.3 CD	4.4 ± 0.2 EFG	<1 G
WSO	6.1 ± 0.4 BCD	1.8 ± 0.2 C	1.1 ± 0.1 C	1.7 ± 0.1 C	5.7 ± 0.4 A	4.0 ± 0.3 A	3.9 ± 0.3 EF	4.0 ± 0.3 AB	6.4 ± 0.4 B	4.0 ± 0.2 BC	5.9 ± 0.4 BC	4.0 ± 0.2 CD
WSP	3.8 ± 0.2 G	0 E	0 D	<1 D	2.5 ± 0.2 D	3.2 ± 0.3 BC	4.0 ± 0.4 DEF	2.9 ± 0.3 D	3.9 ± 0.3 E	2.7 ± 0.2 DE	4.4 ± 0.3 EFG	<1 G
WSQ	3.7 ± 0.2 G	0 E	0 D	<1 D	<1 E	1.2 ± 0.1 HI	1.4 ± 0.1 J	1.7 ± 0.2 F	1.4 ± 0.1 F	1.3 ± 0.1 F	4.1 ± 0.3 FGH	4.2 ± 0.3 BC
WSR	4.0 ± 0.3 G	0 E	0 D	<1 D	<1 E	1.8 ± 0.2 FGHI	2.3 ± 0.3 HI	0 H	3.7 ± 0.3 E	2.0 ± 0.2 EF	4.0 ± 0.2 FGH	2.5 ± 0.2 EF
WSS	6.0 ± 0.3 BCD	0 E	0 D	<1 D	<1 E	1.7 ± 0.2 FGHI	3.9 ± 0.3 EF	1.7 ± 0.1 F	6.1 ± 0.3 BC	3.2 ± 0.3 D	4.2 ± 0.2 FGH	3.8 ± 0.3 CD
WST	6.1 ± 0.3 BCD	0 E	0 D	<1 D	<1 E	2.8 ± 0.2 BCD	5.0 ± 0.3 BC	4.1 ± 0.3 A	6.3 ± 0.2 B	4.6 ± 0.3 B	6.0 ± 0.3 ABC	<1 G

a WSA to -T, wooden shelves from factories A to T, respectively.

b TMM, total mesophilic microorganisms. Results are means ± standard deviations from four plate counts (carried out in duplicates for two independent samplings). Data within a column followed by the same uppercase letter are not significantly different according to Tukey’s tests (*P* < 0.0001).

The four LAB groups (mesophilic and thermophilic rods and cocci) investigated were consistently present in all samples, with the exception of thermophilic cocci on WSI and thermophilic rods on WSA, WSF, WSG, WSI, and WSR. On average, mesophilic cocci were detected at the highest cell densities within the LAB group. The highest levels were observed for the shelf SWH, both for mesophilic rods (8.2 log CFU/cm^2^) and for cocci (8.1 log CFU/cm^2^). In the majority of the shelves analyzed, the levels of mesophilic LAB cocci were comparable to those of TMM, suggesting a dominance of LAB among the members of the wooden shelf viable bacterial community. In the case of WSL, the latter group (6.6 log CFU/cm^2^) was even more represented than TMM (5.3 log CFU/cm^2^). However, the presence of LAB was extremely variable, since low numbers for all four LAB groups were found for the shelves WSF and WSQ or for some of these groups, generally the thermophilic ones. Aside from the group of LAB, enterococci were also enumerated. These bacteria were present in all samples, and their numbers did not exceed 4.1 log CFU/cm^2^; the highest *Enterococcus* cell density was detected in the WSH shelf, the one characterized by the greatest presence of LAB. Regarding members of the *Enterobacteriaceae* family, they were detected in a viable state on only six wooden shelves, while the subgroup of total coliforms was present in four samples. The levels of pseudomonad bacteria were between 2.5 and 5.8 log CFU/cm^2^ in 11 samples and were below the detection limit for the rest of the shelves analyzed.

Filamentous and unicellular fungi were also investigated on the wooden shelves. Molds were not detected in samples from WSA, WSL, WSN, WSP, and WST, and their levels ranged between 1.8 and 5.4 log CFU/cm^2^ in all other shelves. Yeasts developed from all biofilms analyzed ranged from a minimum of 1.8 log CFU/cm^2^ in WSF to 6.8 log CFU/cm^2^ in WSI.

### Microbiological and hygiene criteria for foodstuffs.

Only three shelves presented viable E. coli. In particular, the shelves WSH and WSC were characterized by consistent numbers (3.3 and 4.3 log CFU/cm^2^, respectively). In order to specifically investigate the presence of Shiga-toxigenic E. coli (STEC), the isolates at the highest numbers were processed by PCR to detect STEC genes, and all E. coli cultures from the wooden board biofilms lacked this virulence factor. The presence of coagulase-positive staphylococci (CPS), Salmonella spp., and L. monocytogenes was never found (for this reason, these results are not reported in Table 1), indicating that these pathogens were not present on the wooden boards analyzed.

### Differentiation, identification, and distribution of viable LAB.

Eighty hundred seventy-one colonies were collected from the highest-cell-suspension dilutions of the wooden shelf biofilms plated on the agar media used for LAB counting. A total of 818 isolates were considered presumptive LAB, being Gram positive and unable to split H_2_O_2_ into H_2_O and O_2_. After microscopic inspection, based on morphology and cell arrangement, these cultures were grouped into three main groups: rods, cocci in tetrads, and cocci in short chains. Rod bacteria (39 isolates) constituted barely the minor part of the presumptive LAB community, since the vast majority (779 isolates) of them shared a coccus shape. The combination of all phenotypic features evaluated separated all LAB into only 11 groups (Table 2). LAB rods constituted a single group (I) characterized by a homofermentative metabolism, while cocci were allotted into two groups for tetrads (X and XI) and eight groups (II to IX) for the other bacteria, all forming short chains of cells. Among these, four groups (II to V) were able to generate CO_2_ from glucose, displaying an obligate heterofermentative metabolism. Six groups, including group I, showed thermophilic characteristics, but only the groups VI, X, and XI resisted treatment at 60°C for 30 min. The groups from VI to X were able to grow at high pHs, and the majority of the groups grew under hypersaline conditions.

**TABLE 2:** Phenotypic grouping of LAB isolated from wooden shelf biofilms

Characteristic	Result for cluster^*a*^:										
	1 (*n* = 39)	2 (*n* = 4)	3 (*n* = 7)	4 (*n* = 65)	5 (*n* = 19)	6 (*n* = 46)	7 (*n* = 205)	8 (*n* = 119)	9 (*n* = 266)	10 (*n* = 19)	11 (*n* = 29)
Morphology	R	C	C	C	C	C	C	C	C	C	C
Cell arrangement	sc	sc	sc	sc	sc	sc	sc	sc	sc	tr	tr

Growth at:											
15°C	−	+	+	+	+	+	+	+	+	+	+
45°C	+	−	−	−	−	−	+	+	+	+	+
pH 9.2	ND	−	−	−	−	+	+	+	+	+	−
6.5% NaCl	ND	−	+	+	+	−	+	+	+	+	+

Resistance to 60°C	−	−	−	−	−	+	−	−	−	+	+

Hydrolysis of:											
Arginine	−	−	−	−	−	+	+	+	+	−	−
Esculin	−	−	+	+	+	+	+	+	+	−	−

Acid production from:											
Arabinose	−	+	+	−	+	−	+	+	−	+	+
Ribose	−	−	−	+	+	+	+	+	+	+	+
Xylose	−	−	+	−	+	−	+	−	−	+	+
Fructose	+	+	+	+	+	+	+	+	+	+	+
Galactose	+	−	+	+	+	+	+	+	+	+	+
Lactose	+	−	+	+	+	+	+	+	+	+	+
Sucrose	+	+	+	−	+	+	+	+	−	+	+
Glycerol	+	−	+	+	+	+	+	+	+	+	+

CO_2_ from glucose	−	+	+	+	+	−	−	−	−	−	−

a Abbreviations: R, rod; C, coccus; sc, short chain; tr, tetrads; ND, not determined.

**FIG 3:**
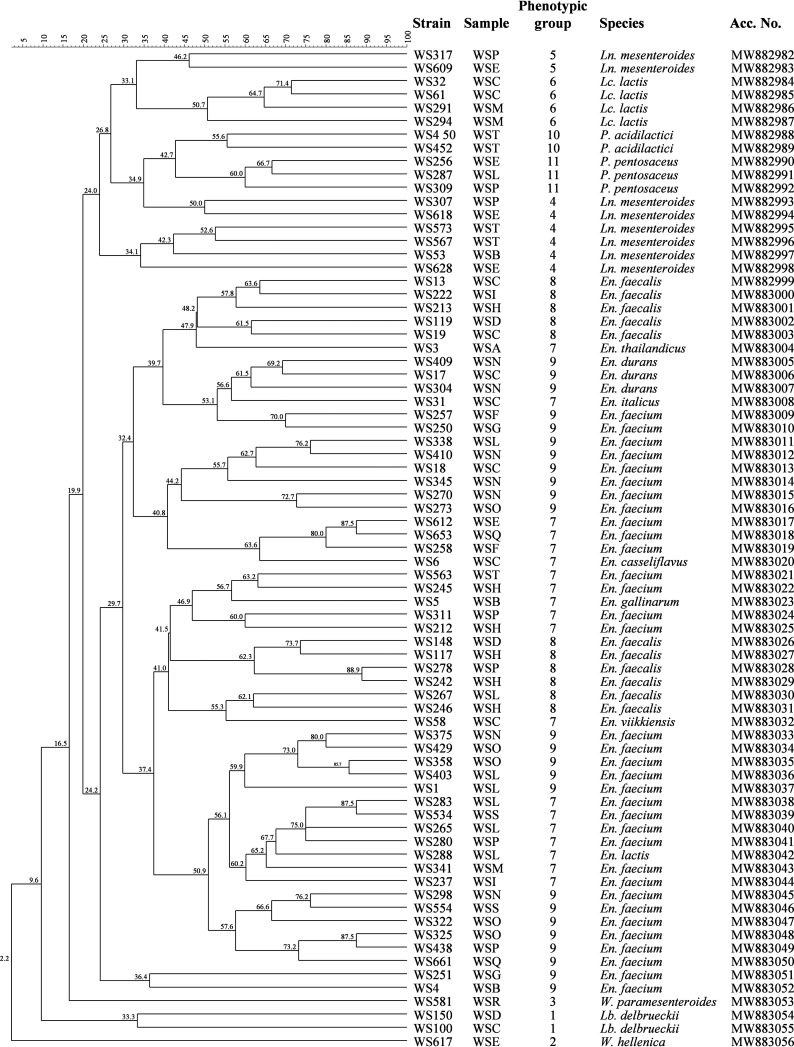
Dendrogram obtained from combined RAPD-PCR patterns generated with three primers of the LAB strains identified from the biofilms on the wooden shelves used to ripen PDO Pecorino Siciliano, PDO Piacentinu Ennese, and Caciocavallo Palermitano cheeses in 18 dairy facilities. Abbreviations: WSA to -T, wooden shelves from factories A to T, respectively; *En.*, *Enterococcus*; *Lb.*, *Lactobacillus*; *Lc.*, *Lactococcus*; *Ln.*, *Leuconostoc*; *P.*, *Pediococcus*; *W.*, *Weissella*; Acc. No., accession number for the 16S rRNA genes.

Within each group, 25% of isolates were taken as representative for the morphological, physiological, and biochemical traits shared by all cultures and genetically differentiated by randomly amplified polymorphic DNA (RAPD) analysis. RAPD patterns were used to construct a dendrogram, and 75 different profiles were recognized among the 205 isolates processed. Thus, the 75 isolates characterized by different RAPD profiles were considered different strains and subjected to 16S rRNA gene sequencing for species identification. All 75 strains were confirmed to belong to the LAB group as *Leuconostoc*, *Lactococcus*, *Pediococcus*, *Enterococcus*, *Lactobacillus*, and *Weissella*. As shown in Fig. 3, the great majority of the strains were allotted into nine species within the genus *Enterococcus*. Almost all enterococci (52 on a total of 54 strains) formed a mega-cluster at ca. 30% homology. In particular, Enterococcus faecalis and Enterococcus faecium were the most numerous species and also showed the highest biodiversity among the members of the wooden shelf viable LAB community. Two *En. faecium* strains (WS4 and WS251) grouped outside the mega-cluster of enterococci. Except *Ln. mesenteroides* strains, which formed two distant clusters, and the two *Weissella* species, which clustered quite far from one another, all other strains grouped by species, such as Lactococcus lactis, and pediococci, which formed distinct clusters. The only two rod-shaped LAB were identified as Lactobacillus delbrueckii and formed a single cluster very distant from the other species.

The dendrogram of Fig. 3 was also useful to perform a speciographic distribution of LAB among the wooden shelves used for cheese ripening. Except for the shelves WSD and WSR, E. faecium was part of the biofilms on all other shelves. E. faecalis was detected in six different biofilms, while the other species were randomly distributed. In particular, the shelves WSF, WSG, WSO, WSQ, SWR, and WSS were characterized by a single viable LAB species, Weissella paramesenteroides, on WSR and E. faecium on the other shelves.

## DISCUSSION

According to the diversity of cheeses reported by McSweeney et al. (25), Caciocavallo Palermitano, PDO Piacentinu Ennese, and PDO Pecorino Siciliano cheeses are all internally bacterially ripened semihard and hard cheeses. These three cheese typologies are mainly characterized by the presence of LAB species during ripening (2, 26, 27). Since they are not inoculated on their surfaces, surface ripening is not considered to occur to the same extent as surface-inoculated smear cheeses, and the rind represents basically a protective barrier for the innermost cheese. For these cheeses, the surface microbiota constitutes a barrier against pathogen and spoilage microorganisms (28). Thus, the wooden shelves used to ripen traditional cheeses of this study were mainly investigated for the safety aspects related to the presence of pathogenic microorganisms.

The wooden shelves of 18 dairy facilities were investigated initially by SEM. This inspection was performed to verify the presence, evaluate the diversity, and observe the type of aggregation of the microbial cells. Microscopic analysis clearly indicated the presence of a mixed bacterial community of cocci and rods on all wooden shelves and showed a general dominance of cocci in the majority of the biofilms investigated. Some yeast cells were also observed, but at a lower frequency than bacteria. Entire coverage by microbial biofilms was also photographed by SEM on the surface of the wooden vats used to transform milk into traditional Sicilian cheeses (10, 15).

Due to the high power of identification and discrimination among microbial populations of next-generation sequencing tools, a high-throughput approach was necessary to investigate deeply the biodiversity of the bacterial biofilms of the wooden shelves used to ripen traditional Sicilian cheeses. *S. equorum* was present in all wooden shelves analyzed, and its percentage covered 2/3 of the total biodiversity. This species, generally found in raw milk and cheeses (29), including smear-ripened cheeses (30), is salt tolerant (31, 32), isolated at high percentages from cheese brine (33), and represents a starter culture component for surface-ripened semihard cheeses (34). The surface-ripening-associated bacteria *Brevibacterium* and *Corynebacterium* (35) were found in almost all wooden shelves, even at 50%. Generally, staphylococci are present at the beginning of smear cheese ripening, while coryneforms characterize the later stages (36). Brachybacteria were also detected in the majority of the biofilms investigated; they represent coryneforms isolated from the cheese surface (37) and have also been isolated from salt-fermented seafoods (38).

Several groups identified belong to halophilic or halotolerant species generally found in the brines used for cheese salting (33). In particular, *Salimicrobium*, *Marinococcus*, *Salegentibacter*, *Haererehalobacter*, *Marinobacter*, and *Idiomarinaceae* are halophilic (39–44), while *Salinicoccus*, *Psychrobacter*, and *Salinisphaera* are moderately halophilic (45–47). All of these species detected on the wooden surfaces of the shelves used for cheese ripening might derive from the sea salt added to the cheese brines (33). Very recently, halophilic bacteria were also detected on the surface of the wooden boards used for cheese ripening in the United States (48).

The LAB community of the wooden shelves included five genera. In general, except for members of *Tetragenococcus*, LAB do not show a high salt tolerance: enterococci, Leuconostoc citreum, and Lactiplantibacillus plantarum (previously known as Lactobacillus plantarum and renamed by Zheng et al. [49]) are reported among the mostly halotolerant LAB (50–52).

Several other bacterial groups were identified, but most of them showed an overall low relative abundance, even though *Bartonellaceae*, *Dietzia*, and *Nesterenkonia* were consistent in a few shelf samples. *Bartonella* spp. are Gram-negative bacteria found in mammalian erythrocytes and endothelial cells transmitted by blood-feeding arthropod ectoparasites (53), probably vectored to the shelves by rodents (54) or mites (55). The genus *Dietzia* includes psychrophilic alkaliphile species (56), while *Nesterenkonia* is another halotolerant genus (57). Members of the *Enterobacteriaceae* family, indicating the presence of fecal bacteria due to fecal contaminations (58), were detected in a very few samples. *A. johnsonii*, reported as a fish pathogen bacterium (59), was found at consistent level only in one sample. Regarding the other populations detected, *Sphingobacteriaceae* are components of the microbiota of different types of soil (60, 61), *Sphingomonadaceae* have different habitats, including seawater (62, 63), and *Alcanivorax* is a marine bacterium.

From a deep comparison with the bacterial composition of the biofilms of the wooden shelves used for the ripening of Fontina cheese (23), a smear-ripened cheese produced in the Valle d’Aosta region of northern Italy, investigated by Illumina technology, it was found that several groups are typically associated with smear cheese production. Hence, based on metagenomics data from the present study, the shelves might transfer to the cheese surface mainly smear-active bacteria, which are responsible for the centripetal maturation of cheese (64), suggesting that the crust of PDO Pecorino Siciliano, PDO Piacentinu Ennese, and Caciocavallo Palermitano cheeses does not play exclusively a passive protective role, but also contributes to the development of the final organoleptic properties of the ripened products.

Although HTS provided a wide and deep bacterial picture of the wooden shelves used to ripen traditional Sicilian cheeses, the plate count technique was applied to estimate the numbers of the viable cells of the main dairy microbial populations. The levels of TMM found on the 18 wooden shelves that were the object of investigation are slightly higher than those reported by Galinari et al. (65) for total aerobic mesophiles on the shelves used for ripening Serro and Canastra cheeses (4.7 and 4.6 log CFU/cm^2^, respectively) produced in Brazil. On the contrary, higher numbers were detected for the total microbial population on the shelves used for the ripening of Reblochon de Savoie smear cheese (>7.0 log CFU/cm^2^) produced in France (16) and for the total bacterial count on the wooden shelves used for Fontina cheese in northern Italy (23). On average, the levels of LAB detected in our study are comparable with those reported by Galinari et al. (65), who found lactococci and lactobacilli in the range 4.0 to 4.7 log CFU/cm^2^ for the wooden shelves of Brazilian cheese ripening. All wooden shelves were also positive for the presence of enterococci in the range 1.1 to 4.1 log CFU/cm^2^. Similar data (2.2 to 4.3 log CFU/cm^2^) were reported by Mariani et al. (16).

In order to better characterize the LAB community, also in view of their possible future application to develop *ad hoc* biofilms on the surface of wooden shelves, the dominant cultures were isolated, phenotypically grouped, genetically differentiated, and identified. LAB cocci represented the majority of isolates. Except for one group including rod-shaped bacteria, LAB included mesophilic, thermophilic, heterofermentative, and homofermentative cocci in linear short chains or tetrads, and some of them were able to grow at high pHs and in the presence of high salt concentrations. The entire community was composed of 75 strains belonging to 16 LAB species. Surprisingly, more than 2/3 of the strains were *Enterococcus*, even though HTS did not reveal their presence. The discrepancy among the results obtained after the isolation procedure and DNA-based Illumina technology could be due to the fact that *Enterococcus* strains were part of the unassigned OTUs of total bacterial community, they were below 0.1% abundance, or their DNAs were rendered inaccessible by nucleases. Similar results were observed also for food matrices (66–68). In particular, nine species within this genus were identified. Basically, the species mostly represented were *En. faecium*, *En. faecalis*, and *Ln. mesenteroides*, but *Lactococcus*, *Pediococcus*, *Lactobacillus*, and *Weissella* were also identified. Considering RAPD patterns, the strains grouped by species, but in some cases, a mixed-species clustering was observed. This trend is common when dairy LAB from wooden biofilms are subjected to a strain typing analysis (13). Most of the species identified from the wooden shelves are generally associated with dairy environments, such as raw milk (69), cheeses (70), animal rennets (71), and wooden vats (13). In particular, Enterococcus viikkiensis is generally identified in ewe’s milk only (72), Enterococcus thailandicus was found in different African dairy traditional fermented products different from cheeses (73, 74) and Weissella hellenica from kimchi (75) a nondairy fermented product.

In light of the Commission Regulation (EC) no. 2073/2005 on microbiological criteria for foodstuffs (14), Salmonella spp. and L. monocytogenes were analyzed by food safety criteria, while E. coli and CPS were analyzed by process hygiene criteria. E. coli was found in only three samples, while CPS, Salmonella spp., and L. monocytogenes were not found in any sample. In general, also the other works on the wooden shelf characterization reported a low frequency of coliforms: in particular, Mariani et al. (16) found this group below 2.2 log CFU/cm^2^, and even lower levels were detected by Galinari et al. (65). Based on the EU One Health 2019 Zoonoses Report (76), STEC infections are the most frequently reported zoonoses after campylobacteriosis and salmonellosis. For this reason, the presence of STEC was specifically investigated in this study. The PCR approach applied clearly indicated that none of the E. coli colonies developed from the wooden board biofilms analyzed in this study were STEC. Fifty-five percent of the samples were characterized by the presence of pseudomonad bacteria (2.5 to 5.8 log CFU/cm^2^). An average presence of 3.0 log CFU/cm^2^ was observed for the shelves used to ripen Reblochon de Savoie smear cheeses (16).

Molds developed from 15 wooden shelves, while yeasts were found in all biofilms analyzed. The presence of these groups is often investigated in cheese, because they play a double role. Regarding yeasts, they might contribute to the centripetal maturation of smear cheeses because they engage in a degradation of lactate, determining a deacidification of the cheese surface where pH increases, stimulating the growth of corynebacteria (77). However, yeasts are also responsible for cheese spoilage, generating undesirable flavor compounds, gas development, surface discoloration, and textural changes (78). If on one hand the role of molds is important to produce soft cheese ripened with surface molds (Camembert and Brie) or to produce blue vein cheeses (Roquefort and Gorgonzola) (79, 80), their contamination can be detrimental to cheese quality—first of all for the generation of potentially toxic secondary metabolites (mycotoxins), but also for their off-flavor and appearance defects (81).

In conclusion, the combined approach applied in this study showed that most bacterial groups identified from the wooden shelves used for the ripening of PDO Pecorino Siciliano, PDO Piacentinu Ennese, and Caciocavallo Palermitano cheeses are typically associated with smear cheeses. Both food safety and process hygiene criteria of EC no. 2073/2005 were almost completely respected; even though E. coli populations were detected on three shelves, they were not STEC strains. Several bacterial taxa belonged to the halophilic/halotolerant group. The effective role of wooden shelf bacteria and fungi has to be more deeply examined in order to better evaluate their contribution to the cheese safety and maturation generating the organoleptic properties at the end of ripening.

## MATERIALS AND METHODS

### Collection of wooden shelf biofilms.

**FIG 4:**
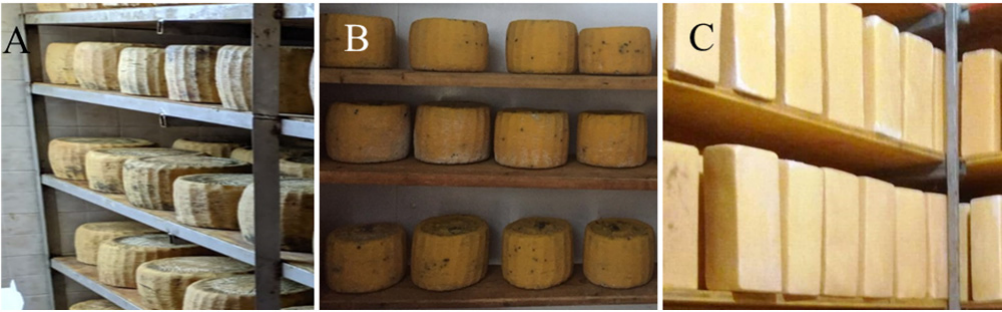
Ripening of traditional Sicilian raw milk cheeses on wooden shelves. (A) PDO Pecorino Siciliano cheese. (B) PDO Piacentinu Ennese cheese. (C) Caciocavallo Palermitano cheese. For ripening, the cheeses are placed in 1 or 2 rows, depending on the width of the board (30 to 60 cm), at a distance of 3 to 5 cm from one another. The length of the boards varies from 2 to 4 m, and the height varies from 3 to 5 cm.

The microbial biofilms associated with the wooden shelves used to ripen three typical Sicilian cheese typologies (PDO Pecorino Siciliano, PDO Piacentinu Ennese, and Caciocavallo Palermitano) (Fig. 4) were collected during ripening from 18 dairy facilities in central and western Sicily within the provinces of Agrigento, Enna, Palermo, and Trapani (Table 3). In particular, the contact of PDO Piacentinu Ennese cheese with the boards lasted 1 to 2 months, while those of the other two cheeses lasted 4 to 5 months. Each facility produces only one cheese type; hence, all wooden boards hosted only one cheese type during ripening. The shelves of all 18 facilities were cleaned once a year, at the end of the summer season, by a thorough brushing followed by being washed with a pressure washer. Square wood splinters of approximately 2 by 2 cm with a thickness of ca. 1 to 2 mm were aseptically cut from each wooden shelf surface with a sterile scalpel (destructive approach) and kept in Falcon tubes. Furthermore, a surface of 100 cm^2^ was delimited on each wooden shelf using a sterile Area Space 100 paper square (VWR International PBI s.r.l., Milan, Italy) and kept under aseptic conditions by a portable Bunsen burner. The microbial populations were collected by the nondestructive brushing technique described by Didienne et al. (11). Briefly, a sterile toothbrush was rubbed vigorously onto the 100-cm^2^ surface, and a complete collection of the microorganisms was obtained with gauze. Subsequently, the toothbrush was shaken into a sterile Durham bottle containing 100 ml of Ringer’s solution (Sigma-Aldrich, Milan, Italy), and the gauze was transferred into the same bottle. Both surfaces sampled by destructive and nondestructive technique were adjacent and collected within the area below the cheeses. Wooden biofilms and splinters were transported by a portable fridge at the laboratory of Agricultural Microbiology of the University of Palermo (Italy). Wooden shelf surface collection was performed in duplicate (technical repeats), considering two cheeses distant approximately 1 m from one another on the same board, and repeated after 1 month for a total of four repetitions in two independent collections.

**TABLE 3:** Characteristics of the wooden shelves used for traditional Sicilian cheese ripening

Wooden shelf^*a*^	City of dairy factory (province)^*b*^	Age of shelf (yr)	Type of wood^*c*^	Cheese typology
WSA	Santo Stefano Quisquina (AG)	16	Silver fir	PDO Pecorino Siciliano
WSB	Santo Stefano Quisquina (AG)	16	Chestnut	Caciocavallo Palermitano
WSC	Castronovo di Sicilia (PA)	15	Silver fir	PDO Pecorino Siciliano
WSD	Godrano (PA)	8	Chestnut	Caciocavallo Palermitano
WSE	Santa Margherita del Belìce (AG)	8	Chestnut	PDO Pecorino Siciliano
WSF	Partanna (TP)	13	Chestnut	PDO Pecorino Siciliano
WSG	Santa Ninfa (TP)	11	Silver fir	PDO Pecorino Siciliano
WSH	Gibellina (TP)	5	Silver fir	PDO Pecorino Siciliano
WSI	Salemi (TP)	8	Silver fir	PDO Pecorino Siciliano
WSL	Aidone (EN)	18	Chestnut	PDO Piacentinu Ennese
WSM	Calascibetta (EN)	7	Chestnut	PDO Piacentinu Ennese
WSN	Assoro (EN)	10	Chestnut	PDO Piacentinu Ennese
WSO	Enna (EN)	1	Swedish pine	PDO Piacentinu Ennese
WSP	Barrafranca (EN)	13	Chestnut	PDO Piacentinu Ennese
WSQ	Godrano (PA)	2	Silver fir	Caciocavallo Palermitano
WSR	Godrano (PA)	4	Chestnut	Caciocavallo Palermitano
WSS	Godrano (PA)	10	Stone pine	Caciocavallo Palermitano
WST	Godrano (PA)	3	Common beech	Caciocavallo Palermitano

a WSA to -T, wooden shelves from factories A to T, respectively.

b Province codes: AG, Agrigento; EN, Enna; PA, Palermo; TP, Trapani.

c Tree species: silver fir, Abies alba L.; chestnut, Castanea sativa Miller; Swedish pine, Pinus sylvestris L.; stone pine, Pinus pinea L.; common beech, Fagus sylvatica L.

### Scanning electron microscopy.

The presence of the biofilms on the surface of the wooden shelves was analyzed by scanning electron microscopy (SEM) using the FEI ESEM Quanta 200 apparatus (FEI Co., Hillsboro, OR, USA) at the Department of Engineering of University of Palermo (Italy). The splinters were dehydrated (82) and dried (10) before being mounted on an aluminum holder. All the samples were sputter coated with a thin layer of gold (83) under argon atmosphere for 90 s (Scancoat Six; Edwards, Crawley, United Kingdom) in order to avoid electrostatic charging under the electron beam.

### Culture-independent microbiological analysis.

Microbial cell suspensions from the wooden shelves (10 ml) were centrifuged at 3,200 × *g* for 15 min at 4°C (26) and total genomic DNAs from biofilms were extracted using the Power Food Microbial DNA isolation kit (Mo Bio Laboratories, Inc., Carlsbad, CA, USA) following the manufacturer’s instructions. No negative control, prepared as a “blank” DNA extraction, was done to check if contaminations were introduced to samples by the DNA extraction method used. In order to prevent the well-to-well contamination (contamination among close processed samples), the single-tube method was preferred to plate-based DNA extraction because the plate-based method was documented (84) as the primary form of well-to-well contamination rather than PCR and single-tube DNA extraction methods. Biofilm DNAs were then purified using the PowerClean DNA Cleanup kit (Mo Bio Laboratories, Inc.) and subjected to quantification through Nanodrop8800 fluorospectrometer (Thermo Scientific, Wilmington, NC) reading. The DNA collected from all 72 biofilms sampled (duplicate samples collected on two distinct collection days per dairy factory) was pooled as four individual wooden shelves per pool in 18 pools representing the 18 dairy factories. A proper balance of the DNA quantity was obtained in each pool to obtain equal representation for each biofilm.

Amplicon library preparation, quality determination and quantification of pooled libraries, and paired-end sequencing using the Illumina MiSeq system (Illumina, USA) were performed at the Sequencing Platform, Fondazione Edmund Mach (FEM), San Michele a/Adige, Italy. Briefly, for each sample, a 464-nucleotide sequence of the V3-V4 region (85, 86) of the 16S rRNA gene (Escherichia coli positions 341 to 805) was amplified. Unique barcodes were attached before the forward primers to facilitate the pooling and subsequent differentiation of samples. To prevent preferential sequencing of the smaller amplicons, the amplicons were cleaned using the Agencourt AMPure kit (Beckman coulter) according to the manufacturer’s instructions; subsequently, DNA concentrations of the amplicons were determined using the Quant-iT PicoGreen double-stranded DNA (dsDNA) kit (Invitrogen) following the manufacturer’s instructions. In order to ensure the absence of primer dimers and to assay the purity, the generated amplicon libraries quality was evaluated by a Bioanalyzer 2100 (Agilent, Palo Alto, CA, USA) using an Agilent high-sensitivity DNA kit. Following the quantitation, cleaned amplicons were mixed and combined in equimolar ratios.

Raw paired-end FASTQ files were demultiplexed using idemp (https://github.com/yhwu/idemp/blob/master/idemp.cpp) and imported into Quantitative Insights Into Microbial Ecology (Qiime2, version 2018.2). Sequences were quality filtered, trimmed, denoised, and merged using DADA2 (87). Chimeric sequences had been identified and removed via the consensus method in DADA2. Taxonomic and compositional analyses were carried on by using the plugins feature-classifier (https://github.com/qiime2/q2-feature-classifier). A pretrained naive Bayes classifier based on the Greengenes 13_8 99% Operational Taxonomic Units (OTUs) database, which had been previously trimmed to the V4 region of 16S rRNA genes, bound by the 341F/805R primer pair, was applied to paired-end sequence reads to generate taxonomy tables.

### Classical microbiological investigation.

Wooden shelf surface biofilms were serially diluted applying a 1:10 dilution factor in Ringer’s solution (Sigma-Aldrich, Milan, Italy). All cell suspensions were then subjected to the plate count technique followed by an appropriate incubation to enumerate total mesophilic microorganisms (TMM), LAB, including enterococci and the main undesired spoilage and pathogenic microorganisms. TMM were detected on plate count agar (PCA) supplemented with 1 g/liter skimmed milk, after aerobic incubation at 30°C for 72 h. Thermophilic and mesophilic LAB cocci were detected on M17 agar, after anaerobic incubation at 30 and 44°C, respectively, for 48 h. Thermophilic and mesophilic LAB rods were detected on de Man-Rogosa-Sharpe (MRS) agar, acidified to pH 5.4 with lactic acid (5 mol liter^−1^), after anaerobic incubation at 30 and 44°C, respectively, for 48 h. Enterococci were detected on kanamycin esculin azide (KAA) agar, after aerobic incubation at 37°C for 24 h. Members of the *Enterobacteriaceae* family were detected on violet red bile glucose agar (VRBGA), after microaerobic incubation at 37°C for 24 h. Coagulase-positive staphylococci (CPS) were detected on Baird Parker (BP) agar with added rabbit plasma fibrinogen, after aerobic incubation at 37°C for 48 h. Pseudomonads were detected on Pseudomonas agar base (PAB) supplemented with cephaloridine sodium fusidate cetrimide (CFC), after aerobic incubation at 25°C for 48 h. Yeasts were detected on dichloran Rose Bengal chloramphenicol (DRBC) agar, after aerobic incubation at 28°C for 48 h. Finally, molds were detected on potato dextrose agar (PDA) supplemented with 0.1 g/liter chloramphenicol to avoid bacterial growth, after aerobic incubation at 25°C for 7 days. In compliance with Commission Regulation (EC) no. 2073/2005 on microbiological criteria for foodstuffs (14), besides CPS, L. monocytogenes, E. coli, and Salmonella spp. were also investigated. L. monocytogenes was detected on *Listeria* selective agar base (LSAB) added with SR0140E supplement, incubated at 37°C for 48 h, while E. coli and Salmonella spp. were detected on Hektoen enteric agar (HEA), incubated at 37°C for 24 h. All media and supplements were purchased from Oxoid (Milan, Italy). Anaerobiosis was realized in hermetically sealed jars containing the AnaeroGen AN25 system (Oxoid). Plate counts were performed in duplicate.

### Isolation and identification of dominant LAB.

In order to characterize the living LAB community of the wooden shelves, Gram-positive (by treatment with 3% [wt/vol] KOH method) and catalase-negative (by treatment with 5% [vol/vol] H_2_O_2_) colonies developed from the highest dilutions of the biofilm cell suspensions plated on MRS and M17 at both incubation temperatures applied were isolated. In particular, at least five colonies sharing the same morphological characteristics (shape, size, margin edge, elevation, color, and opacity of surface) were collected for each colony typology from a given wooden shelf. When less than five colonies developed per morphology, all colonies available were isolated in order to cover the entire wooden shelf LAB diversity. All colonies were transferred into the corresponding broth media and, after overnight development in the optimal incubation conditions, were purified by several streakings onto agar media. Pure cultures were finally developed in liquid media supplemented with 20% glycerol (vol/vol) and stored at −80°C.

After colony differentiation, all isolates were further phenotypically characterized on the basis of cell morphology, growth at 15 and 45°C, resistance at 60°C for 30 min, NH_3_ production from arginine, esculin hydrolysis, acid production from carbohydrates, and CO_2_ production from glucose as reported by Di Grigoli et al. (2). Presumptive LAB cocci were also analyzed for their growth at pH 9.2 and in the presence of NaCl (6.5 g/liter) to preliminarily discriminate enterococci.

In order to perform a differentiation of the wooden shelf LAB at the strain level, a fingerprinting investigation based on the random amplification of polymorphic DNA (RAPD)-PCR analysis was carried out on genomic DNAs extracted from overnight-grown cultures using the InstaGene Matrix kit (Bio-Rad, Hercules, CA, USA) according to the manufacturer’s instructions. Crude cell extracts were amplified using singly the primers M13 (88), AB111, and AB106 (89) under the conditions reported by Settanni et al. (12). RAPD patterns were then analyzed by GelCompar II software version 6.5 (Applied-Maths, Saint-Marten-Latem, Belgium) to obtain a dendrogram and evaluate the similarity among members of the LAB community.

All strains were genetically identified by 16S rRNA gene sequencing performing PCRs as described by Weisburg et al. (90). Sequencing analysis were carried out at the AGRIVET Centre (University of Palermo, Italy) using the ABI Prism 3130xl genetic analyser (Applied Biosystems, Foster City, CA, USA). The resulting sequences were aligned with those present in EzTaxon-e database (http://eztaxon-e.ezbiocloud.net/), which compares a given 16S rRNA gene sequence with only those of the type strains (91). The comparison of the sequences also occurred with 16S rRNA gene sequences available in GenBank/EMBL/DDBJ (http://www.ncbi.nlm.nih.gov). The doubtful identity of *Enterococcus* was resolved by the *sodA* gene-based multiplex PCR assay of Jackson et al. (92).

### Shiga-toxigenic E. coli detection.

In cases where E. coli colonies were detected, they were analyzed for their STEC genes. The multiplex PCR described by Osek (93) and designed on the basis of the genes coding for Shiga toxins 1 and 2 (*stx*_1_ and *stx*_2_) was applied to the E. coli isolates developed at the highest dilutions of the biofilms.

### Statistical analyses.

Plate count data were subjected to one-way analysis of variance (ANOVA) using XLStat software version 7.5.2 for Excel (Addinsoft, New York, NY, USA). The Tukey’s test was applied for pairwise comparison between the different wooden shelf biofilms analyzed. Statistical significance was attributed to *P* values of *P* < 0.05.

### Accession number(s).

The data generated by MiSeq Illumina sequencing were deposited in the NCBI Sequence Read Archive (SRA) and are available under accession no. PRJNA717160 (https://www.ncbi.nlm.nih.gov/bioproject/717160). All sequences determined in this study were deposited in GenBank database under accession no. MW882982 to MW883056.
